# A *Photorhabdus* Natural Product Inhibits Insect Juvenile Hormone Epoxide Hydrolase

**DOI:** 10.1002/cbic.201402650

**Published:** 2015-02-25

**Authors:** Friederike I Nollmann, Antje K Heinrich, Alexander O Brachmann, Christophe Morisseau, Krishnendu Mukherjee, Ángel M Casanova-Torres, Frederic Strobl, David Kleinhans, Sebastian Kinski, Katharina Schultz, Michael L Beeton, Marcel Kaiser, Ya-Yun Chu, Long Phan Ke, Aunchalee Thanwisai, Kenan A J Bozhüyük, Narisara Chantratita, Friedrich Götz, Nick R Waterfield, Andreas Vilcinskas, Ernst H K Stelzer, Heidi Goodrich-Blair, Bruce D Hammock, Helge B Bode

**Affiliations:** [a] Merck Stiftungsprofessur für Molekulare Biotechnologie, Fachbereich Biowissenschaften, Goethe Universität Frankfurt 60438 Frankfurt am Main (Germany) E-mail: h.bode@bio.uni-frankfurt.de; [b] Department of Entomology and Nematology & UCD Comprehensive Cancer Center, University of California One Shields Avenue, Davis, CA 95616 (USA); [c] Department Bioresources, Fraunhofer Institute for Molecular Biology and Applied Ecology (IME) Winchesterstrasse 2, 35394 Giessen (Germany); [d] Department of Bacteriology, University of Wisconsin–Madison 1550 Linden Dr,. Madison, WI, 53706 (USA); [e] Institute for Cell Biology and Neuroscience and Buchmann Institute for Molecular Life Sciences (BMLS), Goethe Universität Frankfurt 60438 Frankfurt am Main (Germany); [f] Cardiff School of Health Sciences, Cardiff Metropolitan University Llandaff Campus, Western Avenue, Cardiff, CF5 2YB (UK); [g] Swiss Tropical and Public Health Institute, Parasite Chemotherapy, University of Basel Socinstrasse 57, 4051 Basel (Switzerland); [h] Microbial Genetics, Interfaculty Institute of Microbiology and Infection Medicine, University of Tübingen Auf der Morgenstelle 28, 72076 Tübingen (Germany); [i] Vietnam National Museum of Nature, Vietnam Academy of Science and Technology 18 Hoang Quoc Viet, Cau Giay, Hanoi (Vietnam); [j] Department of Microbiology and Parasiology, Faculty of Medical Science, Naresuan University 99 Moo 9 Phitsanulok-Nakhon Sawan Road, Tha Pho Mueang Phitsanulok, 65000 Phitsanulok (Thailand); [k] Department of Microbiology and Immunology, and Mahidol-Oxford Tropical Medicine Research Unit, Faculty of Tropical Medicine Bangkok 10400 (Thailand); [l] Division of Translational and Systems Medicine, Unit of Microbiology and Infection, Warwick Medical School, University of Warwick Coventry, CV4 7AL (UK); [m] Buchmann Institute for Molecular Life Sciences (BMLS), Goethe Universität Frankfurt 60438 Frankfurt am Main (Germany)

**Keywords:** biosynthesis, entomopathogenic bacteria, juvenile hormone epoxide hydrolase inhibitor, natural products, *Photorhabdus*

## Abstract

Simple urea compounds (“phurealipids”) have been identified from the entomopathogenic bacterium *Photorhabdus luminescens*, and their biosynthesis was elucidated. Very similar analogues of these compounds have been previously developed as inhibitors of juvenile hormone epoxide hydrolase (JHEH), a key enzyme in insect development and growth. Phurealipids also inhibit JHEH, and therefore phurealipids might contribute to bacterial virulence.

## Introduction

Natural products have been used in medicine since ancient times, and especially in the past 70 years they have served us well as anti-infective, anticancer and other therapeutics.[1, 2] Despite their great benefit to human health it is mostly unknown why nature has developed these compounds and what their biological roles are,[3, 4] although examples of natural products acting as virulence factors,[5] signalling molecules[6] and antimicrobials[7] are known. Entomopathogenic bacteria of the genus *Photorhabdus* live in symbiosis with nematodes of the genus *Heterorhabditis*, and together they are able to infect and kill insect larvae. Probably because of the complex bacterial interactions with the nematode host and the insect prey (communication within the bacterial community and between bacteria and nematodes, virulence against the insect prey, defence against food competitors) these bacteria are producers of several natural products.

Here, we describe urea lipid compounds, which we name “phurealipids” (*Photorhabdus* urea lipids) produced by the insect pathogen *Photorhabdus luminescens* to inhibit juvenile hormone epoxide hydrolase (JHEH), a key enzyme in insect development and growth; similar compounds have been developed chemically as insecticides.

## Results and Discussion

**Figure 1:**
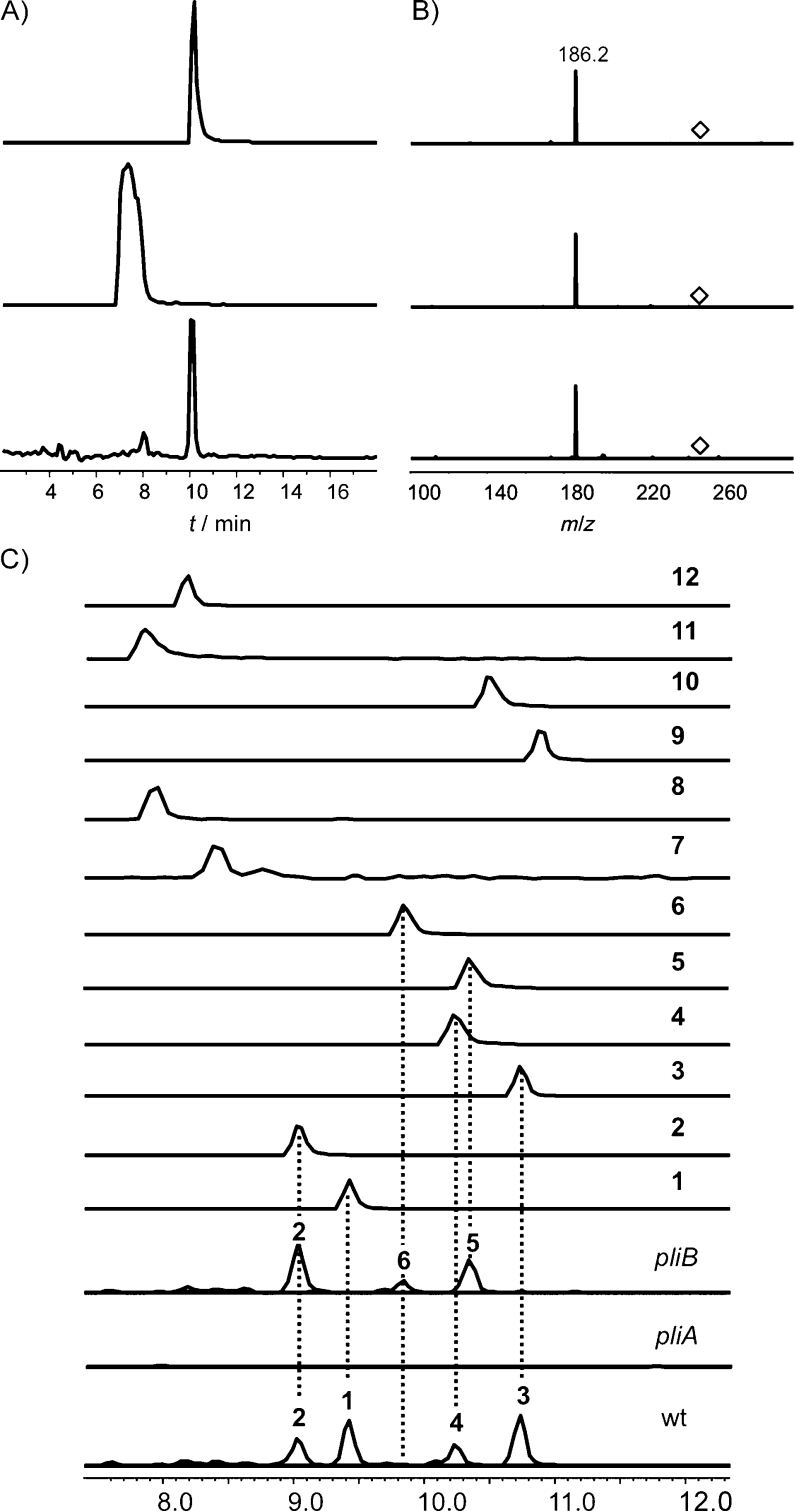
A) Extracted ion chromatograms (*m*/*z* 243.2), and B) MS/MS analysis of synthetic 4 (top), the corresponding glycine amide (middle) and natural 4 (bottom); diamond: mother ion. C) Extracted ion chromatograms of the natural phurealipids 1–6 from *P. luminescens* TTO1 (wt and *pliA* and *pliB* mutants) in comparison with the synthesised compounds: *m*/*z* 229.2 (1, 6 and 7), *m*/*z*.215.2 (2 and 8), *m*/*z* 257.2 (3 and 9), *m*/*z* 243.2 (4, 5 and 10), *m*/*z* 201.2 (11) and *m*/*z* 187.2 (12). The dotted lines highlight identical retention times between natural and synthetic compounds. Disruption of *pliA* led to total loss of phurealipid production.

**Figure 2:**
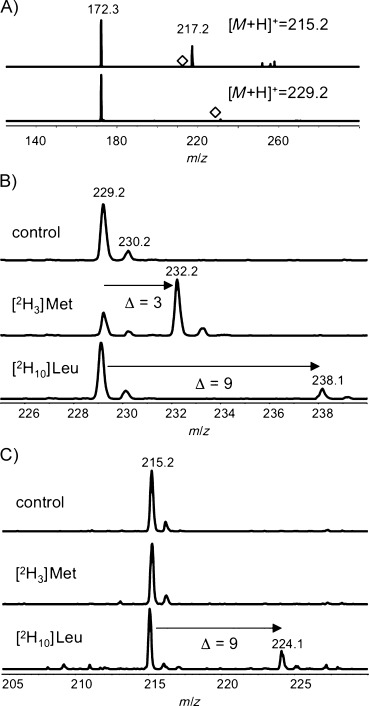
A) MS^2^ data of 1 (bottom) and 2 (top). MS data of B) 1 and C) 2 obtained from labelling experiments in strain TTO1 (control with no additives, addition of l-[methyl-^2^H_3_]methionine and l-[2,3,3,4,5,5,5,6,6,6-^2^H_10_]leucine (from top to bottom)).

A detailed HPLC/MS analysis of *P. luminescens* TTO1 showed the presence of four compounds (**1**–**4**) with *m*/*z* between 215 and 257 [*M*+H]^+^ (Table S1 in the Supporting Information). The molecular formulae of **1**, **3** and **4** as determined by HR-ESI-MS (Table S1) in addition to their mass fragmentation patterns indicated either a glycine amide or a urea-derived structure; the loss of 57 Da is characteristic for either a glycine or a methyl urea moiety (Figure 1). The structure and nature of the alkyl side chains were confirmed by labelling experiments (Table S1). Briefly, **1**–**3** were labelled with fully deuterated leucine, thereby indicating the presence of a leucine-derived iso-branched fatty acid; no labelling was observed with deuterated valine (indicative of *iso* even branched amines) or propionic acid (indicative of uneven linear amines) for any compound. A linear, even-numbered side chain similar to that from standard fatty-acid biosynthesis was assumed for **4**. Compounds **1**, **3** and **4** showed the expected labelling with deuterated l-[methyl-^2^H_3_]methionine exclusively at the polar moiety and not the side chain (where it could also occur following the biosynthesis of methylated fatty acids), thus confirming a methyl urea moiety (Table S1). This was subsequently proven by chemical synthesis of both glycine amide and the methyl urea derivative of **4** (identical *t*_R_ values for synthetic and natural **4**). Compound **2** showed a neutral loss of 43 Da, corresponding to the desmethyl derivative of **1** (Figure 2).

**Scheme 1:**
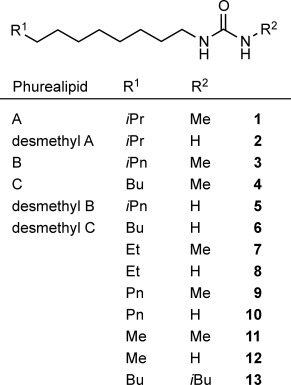
Natural phurealipids 1–6 and synthetic derivatives 7–13.

**Scheme 2:**
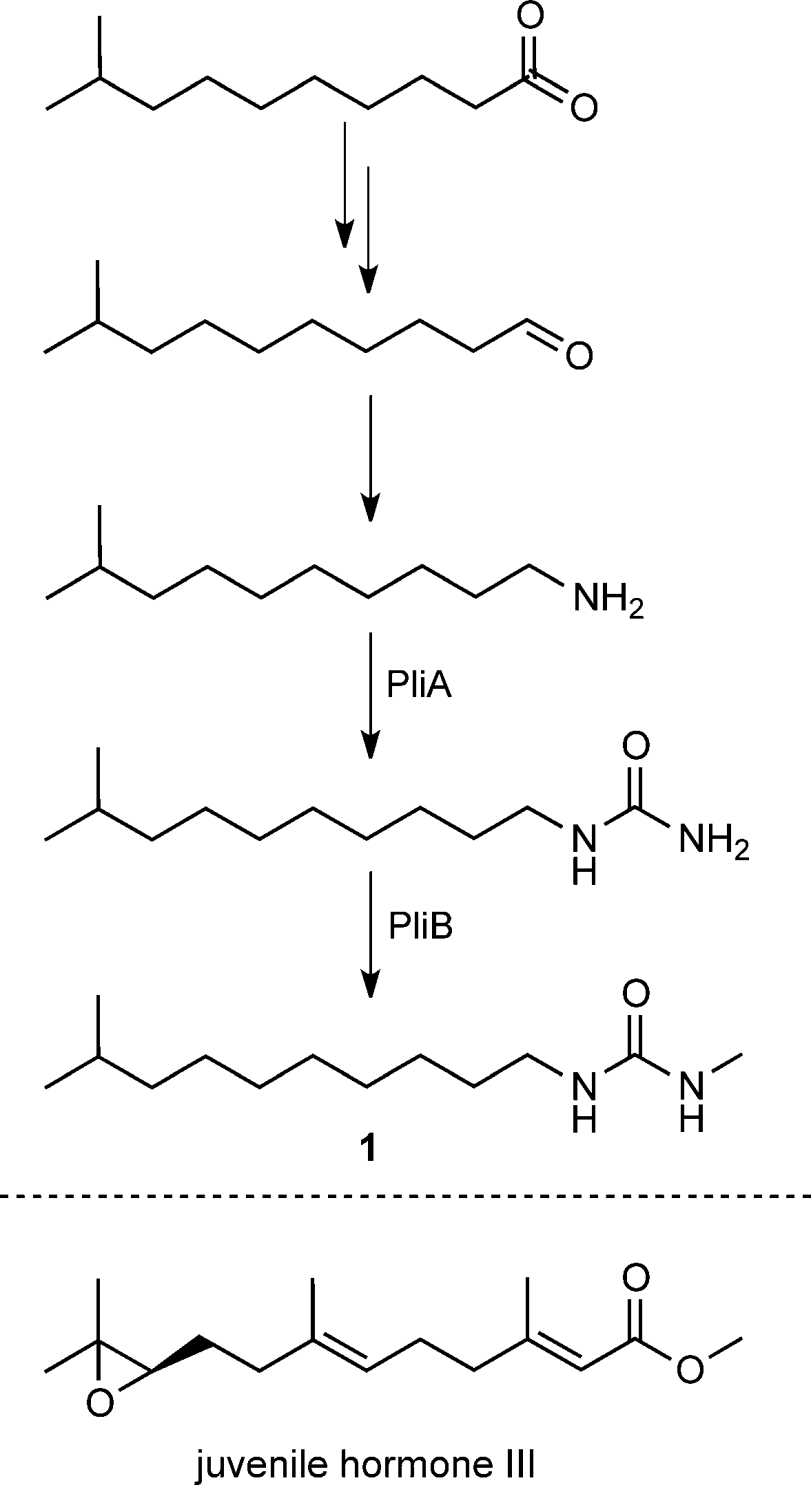
Proposed biosynthesis of phurealipid A (1), and structure of JH III.

**Figure 3:**
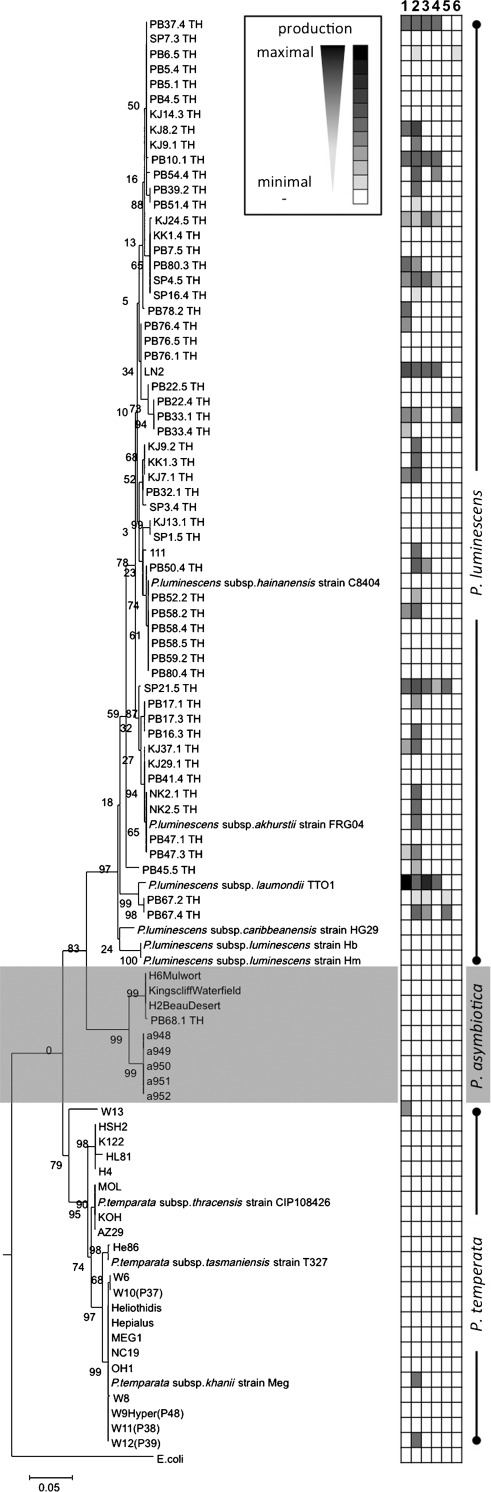
Phylogenetic tree based on a 646 bp region of *recA* (encoding the highly conserved RecA protein involved in DNA repair) for different *Photorhabdus* strains (outgroup: *E. coli*).[34] The tree was reconstructed by the maximum likelihood approach (ClustalW alignment). Jukes-Cantor (JC69) was used as substitution-model; bootstrap values are based on 1000 replicates. Right: relative production of phurealipids 1–6. All strains were analysed by HPLC/MS; mostly strains of *P. luminescens* produce phurealipids, as identified by retention time and MS/MS data.

Based on the structures of the identified phurealipids (Scheme 1), a biosynthetic pathway was postulated starting from different fatty-acid-derived aldehydes, which are subsequently transformed into the corresponding amines, carbamoylated and finally methylated (Scheme 2). Two carbamoyltransferases were identified in the genome of the producing strain. Gene disruption by plasmid integration (Figure S1 in the Supporting Information) into one of them, *plu2076* (here renamed *pliA* (phurealipid)), led to complete loss of phurealipid production. Disruption of the second carbamoyltransferase, *plu4565*, did not affect phurealipid biosynthesis, although these mutants were no longer able to produce a virulence factor that we termed “*Photorhabdus* clumping factor” or “PCF”,[8] the structure of which is currently unknown. Despite the fact that more than 15 methyltransferase homologues were identified in the *P. luminescens* genome, comparative genome analysis between different *Photorhabdus* and *Xenorhabdus* strains revealed only *plu2237* to be unique to *P. luminescens* (the only phurealipid producer with a sequenced genome).[9] Subsequent gene disruption (Figure S1) of *plu2237* (which we renamed *pliB*) led to the biosynthesis of a different phurealipid profile. Detailed MS and labelling experiments revealed the presence of desmethylphurealipids B (**5**) and C (**6**; Figure 1, Table S1), whose structures were confirmed by synthesis. A search for additional phurealipid-producing strains in our entomopathogenic bacteria strain collection[10] based on HPLC/MS analysis of over 250 strains revealed **1** to be widespread in *P. luminescens* strains (Figure 3, Figure S2) but very rare in *Photorhabdus asymbiotica* or *Photorhabdus temperata*, consistent with the fact that no *plu2076* homologue could be found in the genome of *P. asymbiotica*.[11] However, three *Xenorhabdus* strains isolated in Vietnam and related to *Xenorhabdus ehlersii* DSM 16337 showed production of **1** (Figure S3).

In independent research, closely related synthetic compounds have been previously described as inhibitors of insect juvenile hormone epoxide hydrolase (JHEH).[12]–[14] In conjunction with juvenile hormone esterase (JHE), JHEH is a key player in the degradation of juvenile hormone (JH), which regulates both growth and development of insect larvae and reproductive functions of adults,[15] and is also produced by the plant *Cyperius iria* as a defence mechanism against insects.[16] Importantly, *P. luminescens* phurealipids and the related synthetic insecticides are structurally similar to JH (Scheme 2), thus suggesting a possible mode of action. We tested all phurealipids against JHEH purified from caterpillars of the tobacco hornworm *Manduca sexta* and demonstrated that **1**, **3** and **4** showed IC_50_ values of 6.5±0.9, 30±4, and 10.7±1.2 μm, respectively. These are in a similar range to that observed for the known synthetic inhibitor **13** (Scheme 1, Table S2; IC_50_=2.3±0.6 μm) and is in agreement with comparable *K*_I_ values (1.80±0.30 and 0.35±0.04 μm for **1** and **13**, respectively; Figure S4). Although desmethylphurealipid B (**5**) showed weak activity against JHE (IC_50_=25±4 μm), no other derivatives showed activity (>100 μm) against either JHE or JHEH (Table S2).

Upon infection of *Galleria mellonella* larvae, *P. luminescens* produced phurealipids at up to 200 μm (≈44 mg L^−1^; Figure S5) as determined by HPLC/MS. This would be sufficient to inhibit JHEH and thus might lead to an increase in JH. JH accumulation in *Drosophila melanogaster* inhibits the production of antimicrobial peptides (AMPs), thus indicating that JH acts as a humoral immuno-suppressor.[17] Hence, manipulation of JH levels influences not only insect development but also the efficacy of the immune response. Taken together, these data suggest that phurealipids contribute to the overall virulence of *P. luminescens* by inhibiting JHEH activity and therefore limiting AMP production.

To test this hypothesis, we used quantitative reverse-transcriptase PCR to measure the RNA levels of certain AMP genes (lysozyme, gallerimycin, moricin and cecropin) in caterpillars of *M. sexta* and the greater waxmoth *G. mellonella* challenged with *Serratia entomophila* or *Salmonella enterica*, respectively, following injection of different urea lipid compounds (Figure S6). The known synthetic inhibitor **13** demonstrated the best activity (lower levels of AMP RNAs relative to the control). Of the natural compounds, desmethylphurealipid A (**2**) was the most active in this assay but showed no JHEH inhibitory activity in vitro, thus suggesting other or additional JHEH independent activities for phurealipids in vivo.

We also tested whether urea lipids predicted to lead to JH III accumulation by JHEH inhibition influence the embryonic development of the emerging insect model organism *Tribolium castaneum*. As methoprene was described[18] as a JH III mimic used as insecticide, it was used as positive control for **13** (the most active urea compound inhibiting JHEH). All *T. castaneum* embryos treated with methoprene proceeded through gastrulation, germ band elongation and germ band retraction normally, but failed to internalise the remaining yolk sac during dorsal closure (Figure S7; Supporting Movie 1). The result was significant compared to the PBS and DMSO controls (Figure S7 c), thus confirming the insecticidal effect of this compound.[19, 20] In contrast, embryos treated with **13** were able to proceed through dorsal closure normally (Figure S7, Supporting Movie 1), and the percentage of embryos successfully completing development did not differ significantly from those subjected to PBS and DMSO (Figure S7).

JH has been reported to influence gene expression in protozoan termite gut symbionts[21] and to play a role in Ca^2+^ homeostasis,[22] in addition to exerting epigenetic control of gene expression.[23] Based on their structural resemblances, similar activities might be exist for phurealipids. Indeed, the desmethyl urea lipids in particular exhibited very strong activity against *Leishmania donovani* and were in fact at least 10 times more active than the methylated derivatives (Table S2). *L. donovani*, the causative agent of leishmaniasis (kala-azar), is not known to employ any JH-like regulatory pathways, but the promising activity of such simple compounds will be studied in more detail in the future. Further bioactivity tests revealed neither antibacterial nor antifungal activity for any phurealipid, and although other urea derivatives are quorum quenching compounds in Gram-negative bacteria,[24] no such activity was observed for phurealipids. In experimental infections of the caterpillar *M. sexta*, no difference in virulence was observed between the *pliA* insertion mutant and the parental wild-type strain. Nevertheless, because of the large redundancy of virulence factors in this bacterium it is likely that the contribution of the phurealipids to the overall virulence is masked. Precedence for this can be seen in the observation that strains lacking the highly potent Mcf1 toxin remain as virulent as the wild type,[25] and disruption of rhabdopeptide biosynthesis in the related bacterium *Xenorhabdus nematophila* had only a slight effect on overall toxicity whereas the pure compounds showed insecticidal activity.[26] Moreover, it has been proposed that the “stacking” of multiple virulence factors gives *P. luminescens* a selective advantage during typical suboptimal infection scenarios and in diverse hosts in nature.[25]

It is interesting that *P. luminescens* produces a small library of phurealipids in similar amounts, as has been observed for other compound classes, such as rhabdopeptides, xentrivalpeptides and taxlllaids.[26]–[28] Because the nematode vector shows little insect host specificity,[29] we propose that this might provide *P. luminescens* with the ability to inhibit diverse JHEHs from a range of insect orders. JHs differing in the presence of methyl or ethyl substituents, degree of saturation and epoxide moieties have been described from different insects, thus making the presence of slightly different JHEHs quite likely.[30]

The phurealipids offer a rare example of a compound class originally developed by synthetic chemists to address a specific molecular target, but which had in fact already been “developed” by *P. luminescens* much earlier. We can draw parallels with the cultivation and use of antifungal-producing *Streptomyces* by leaf-cutting ants[31, 32] or bark beetles[33] to protect their fungal gardens against pathogenic fungi, similar to humans using such compounds in antifungal therapy. These examples clearly highlight the value of organisms producing natural product, both as sources of molecules and as inspiration for much-needed novel bioactive compounds.

## Supporting Information

As a service to our authors and readers, this journal provides supporting information supplied by the authors. Such materials are peer reviewed and may be re-organized for online delivery, but are not copy-edited or typeset. Technical support issues arising from supporting information (other than missing files) should be addressed to the authors.

miscellaneous_information
