# Supplementary material for: A Photorhabdus Natural Product Inhibits Insect Juvenile Hormone Epoxide Hydrolase
Source: Chembiochem. 2015 Feb 25;16(5):766–71. doi: 10.1002/cbic.201402650 (PMC4486325; doi:10.1002/cbic.201402650)
Supplement: Supplementary file 2 — miscellaneous_information [file cbic0016-0766-sd2.pdf]

CHEMBIOCHEM

Supporting Information

A *Photorhabdus* Natural Product Inhibits Insect Juvenile Hormone Epoxide Hydrolase

Friederike I. Nollmann,[a] Antje K. Heinrich,[a] Alexander O. Brachmann,[a] Christophe Morisseau,[b] Krishnendu Mukherjee,[c] Ángel M. Casanova-Torres,[d] Frederic Strobl,[e] David Kleinhans,[e] Sebastian Kinski,[a] Katharina Schultz,[a] Michael L. Beeton,[f] Marcel Kaiser,[g] Ya-Yun Chu,[h] Long Phan Ke,[i] Aunchalee Thanwisai,[j] Kenan A. J. Bozhüyük,[a] Narisara Chantratita,[k] Friedrich Götz,[h] Nick R. Waterfield,[l] Andreas Vilcinskas,[c] Ernst H. K. Stelzer,[e] Heidi Goodrich-Blair,[d] Bruce D. Hammock,[b] and Helge B. Bode*[a, m]

cbic_201402650_sm_miscellaneous_information.pdf
cbic_201402650_sm_miscellaneous_information.avi

Supplementary Material

Methods

General. Cultivation of *Photorhabdus luminescens* TT01 and its plasmid integration mutants plu2237::cat and plu2076::cat was performed as described previously [1-3] with the appropriate antibiotics in the following concentrations: ampicillin 100 µg/ml, chloramphenicol 20 µg/ml, rifampicin 50 µg/ml. Natural product analysis by HPLC-MS/MS and HRESI-MS/MS [4-6], natural product quantification in LB and *Galleria mellonella* [3], and inhibition of JHEH [7] and JHE [8,9] from *Manduca sexta* were performed as described previously. Bioactivity against *Escherichia coli*, *Micrococcus luteus*, *Bacillus subtilis*, *Saccharomyces cerevisiae* was performed as described [10]. Quorum quenching assays have also been described previously [11]. Phylogenetic analysis of *Photorhabdus* was performed as described [12] and all strains listed in Figure 4 were cultivated and analyzed by HPLC/MS as described previously [2,3,6]. Feeding experiments [3] were performed as follows: L-[2,3,4,4,4,5,5,5-$^2H_8$]valine, L-[2,3,3,4,5,5,5,5′,5′,5′-$^2H_{10}$]leucine, L-[*methyl*-$^2H_3$]methionine and [2,2,3,3,3-$^2H_5$]propionic acid were fed to LB medium with Amberlite XAD-16. 5 mL of LB media with 2% XAD-16 in 50 mL Erlenmeyer flasks were inoculated to an OD_{600} of 0.1 with a preculture in the same medium without XAD16. Possible precursors were fed at 0, 24 and 48 h after inoculation to a final concentration of 2 mM. Cultures were harvested after 72 h of cultivation at 30 °C and 180 rpm. To obtain the crude extracts the XAD beads were separated from the supernatant by decanting and for 1 h extracted with 5 ml MeOH. The metabolites in the crude extracts were identified using HPLC/MS analysis.

Analysis of phurealipid production. *Galleria mellonella* larvae were used for the determination of the phurealipid production in insects as described previously [3]. The experiment was performed in duplicates and covered a time period of 14 days, samples were taken after 6h, 12h, 24h, 36h, 48h, 3d, 4d, 7d, 10d, and 14d. Each sample comprised ten

larvae that were infected with a suspension of *P. luminescens* TT01 cells. For this, an overnight culture of TT01 was washed with LB and adjusted to an optical density of OD_{600} 1.0 and each larva was injected by syringe in the midabdominal proleg with 5 µl of cell suspension. Aseptic LB medium was used as a control. All infected larvae were dead after 48 hours, while 95% of the control group survived. Each sample was snap-frozen in liquid nitrogen. The frozen larvae were ground to a crude powder using a pestle and mortar. The powder was extracted for 1 hour with 50 mL of a methanol/acetone (1:1) solvent mixture, filtered and evaporated. The crude extract was re-dissolved in 3 mL of methanol and diluted 1:10 before HPLC-MS analysis. Production kinetics were analysed using triplicates in LB broth. *P. luminescens* TT01 was cultivated in 1 L Erlenmeyer flasks containing 100 mL LB using the described cultivation conditions. For each time point 4 mL of culture broth was extracted with the same solvent mixture, filtered and the solvents removed under reduced pressure. The crude extract was then dissolved in 1 mL methanol and without further dilution submitted to HPLC-MS analysis.

Construction of the carbamoyl transferase and the methyl transferase mutants. Carbamoyl transferase and methyl transferases encoded in the genome of *P. luminescens* TT01 [13] were identified using their homology to known enzymes from *E. coli* and by comparison between all *Xenorhabdus* [14,15] and *Photorhabdus* [13,16] genomes currently available. The genes *plu2076* and *plu2237* were only present in *P. luminescens* and encode a carbamoyl transferase and a methyl transferase, respectively. They were disrupted by plasmid integration as described previously. Briefly, the suicide vector pDS132 [17] or pCKcipB (a pDS132 derivative with an additional *Bgl*II recognition site) both carrying a chloramphenicol resistance gene (cat) were used. An internal fragment of *plu2237* (*pliB*) was amplified with the following PCR primers: KO_plu2237_PstI_fw (5′-ATAT**CTGCAG**CATATGGAATTTCCACAAGAGG-3′) containing a *Pst*I restriction side

(bold) and KO_plu2237_BamHI_rev (5′-ATAT**GGATCC**GAATCTTTTGGTTATAATCAAGAGC-3′) containing a *Bam*HI restriction side (bold), yielding a product of 534 bp. This amplicon was subcloned into vector pJET1.2 (Thermo Scientific, Fermentas) and subsequently digested with the restriction endonucleases *Pst*I and *Bam*HI (both Fermentas). This restriction fragment was ligated into pCKcipB linearised with *Pst*I and *Bgl*II. The resulting plasmid pDS2237KO was transformed by electroporation into *E. coli* S17-1 λpir (Tpr Smr *recA thi hsdR* RP4-2-Tc::Mu-Km::Tn7, λpir phage lysogen). Subsequently this construct was conjugated into a rifampcin-resistant TT01 strain as previously described [1].

The plasmid donor strain and the acceptor strain were grown to an OD_{600} of 0.6-0.8 and spotted together (in a ration of 1:3) onto a LB agar plate without any selection marker. Cells were incubated at 37°C for 3 h and another 21 h at 30°C. The cell lawn was harvested using an inoculation loop and resuspended in 2 ml LB medium. For selection of TT01 colonies with acquired chloramphenicol resistance different volumes of the cell suspension (50 µl, 100 µl and 200 µl) were plated on LB agar plates with rifampicin and chloramphenicol. The genotype of the chloramphenicol resistant strain TT01-2237::cat was confirmed by PCR using a plasmid-specific pair of primers pDS132fw (5'-GATCGATCCTCTAGAGTCGACCT-3') and pDS132rv (5'-ACATGTGGAATTGTGAGCGG-3') in combination with a genome-specific pair of primers V_KO_plu2237_fw (5′-GAGCGATTCTGGCTAAATCG-3′) and V_KO_plu2237_rev (5′-TTGATGTTTACGGCGATGG-3′).

For the generation of the *plu2076* (*pliA*) insertion mutant in *P. luminescens* the same approach was applied: An internal fragment of plu2076 was amplified with the primers KOplu2076SacI (5′-AATGCT**GAGCTC**ACGTTTTCTGCGAGAGAATAATCCA-3′) containing a *Sac*I restriction side (bold) and KOplu2076PaeI (5′-ATATTC**GCATGC**CATTATCAAATGCTGGCGGTAAC-3′) containing a *Pae*I restriction side (bold) which yielded a product of 740 bp that was then subcloned into vector pJET1.2

and subsequently cloned into pDS132 via the *Sac*I and *Pae*I restriction sites. The resulting plasmid pDS2076KO was introduced into *E. coli* S17-1 λpir by electroporation and the *P. luminescens* plasmid insertion mutant TT01-2076::cat was generated as described. The correct plasmid insertion was confirmed by PCR using again the plasmid-specific primers pDS132fw and pDS132rv as well as genomic-specific primers V2076_Fw (5′-TTCGCTAGAAGCACCGCATT-3′) and V2076_Rv (5′-TCAGCAAAGTTGCGCAAAGC-3′).

Comparison of *in vivo* virulence of *P. luminescens* WT and *pliA* mutant. *P. luminescens* wt and *pliA* mutant were grown overnight in LB broth with shaking at 28°C in the dark. Sub-cultures were grown to mid exponential phase (OD_{600} = 0.2-0.4). Dilutions of bacteria where then injected into first day 5th instar *Manduca sexta* larvae, which were reared as previously described [18]. Briefly, larvae were maintained individually at 25˚C under a photoperiod of 17 hours light: 7 hours dark and fed on an artificial diet based on wheat germ. Unless otherwise stated, larvae 1 day after ecdysis to the 5th instar were used for all experiments.

For the two strains approximately 100, 1000 and 10000 viable cells (confirmed by overnight plating) were injected into a cohort of 12 animals each, which were then monitored for mortality (movement and turgidity) by regular physical stimulus over 3 days and again at 7 days.

Determination of antimicrobial peptide transcript levels in *Galleria mellonella* and *Manduca sexta*. Stock solutions of the different phurealipids were prepared by dissolving the compounds separately in 100% ethanol. For injection, 1:100 dilutions of individual compound were used for injection. The concentrations injected were as follows: **1** (46 µg/ml), **2** (28 µg/ml), **3** (28 µg/ml), **4** (31 µg/ml), **5** (28 µg/ml), **6** (28 µg/ml), **7** (22 µg/ml), **8** (28 µg/ml), **9** (28 µg/ml), **10** (28 µg/ml), **12** (28 µg/ml), and **13** (44 µg/ml).

Larvae of *G. mellonella* were reared at 32°C in darkness and on an artificial diet (22% maize meal, 22% wheat germ, 11% dry yeast, 17.5% bees wax, 11% honey, 11% glycerin). Last-instar larvae, each weighing 250–350 mg, were used in all experiments as described previously [19-21].

To induce immune responses in *G. mellonella* larvae were injected with *t*he entomopathogenic bacterium *Serratia entomophila* obtained from the DSMZ (Deutsche Sammlung von Mikroorganismen und Zellkulturen GmbH) and cultured aerobically in LB medium at 37°C. Overnight cultures were diluted (1:50), and grown till OD-1.0. *S. entomophila* in its exponential phase (10^9 cfu/ ml in 10 ml LB broth) were washed and serially diluted with 1x PBS. For each insect, 10 µl of culture (10^6 cfu/larva) was injected dorsolaterally into the hemocoel of last-instar larvae using 1-ml disposable syringes and 0.4 × 20 mm needles mounted on a micromanipulator. After 1.5 h infected larvae of *G. mellonella* were injected with urea lipid compounds (15 µl/larva). Samples were collected for RNA isolation after incubation for 2 hours at 37°C. The control larvae previously injected with *S. entomophila* were further challenged with 1% ethanol.

For quantitative reverse transcriptase (qRT) PCR in *G. mellonella,* five larvae per injected phurealipid were homogenized in 1 ml Trizol reagent (Sigma Aldrich), and whole RNA was extracted according to the manufacturer's recommendations. Complementary DNA (cDNA) synthesis was performed using the First Strand cDNA synthesis kit (Fermentas). The RNA and cDNA quantity was spectrophotometrically determined and the integrity was confirmed by ethidium bromide gel staining. Quantitative real time RT-PCR was performed with the Biorad real-time PCR system (CFX 96) using the SsoFast EvaGreen Supermix protocol (Biorad). We used 50 ng of cDNA per reaction to amplify genes like moricin, gallerimycin

and lysozyme using the following primers: Gallerimycin-forward (5’-CGCAATATCATTGGCCTTCT-3’) and Gallerimycin-reverse (5’-CCTGCAGTTAGCAATGCACTC-3’), Lysozyme-forward (5’-TCCCAACTCTTGACCGACGA-3’) and Lysozyme-reverse (5’-AGTGGTTGCGCCATCCATAC-3’), Moricin-like peptide A-forward (5’-GCGATCATTGCCCTCTTTAT-3’) and Moricin-like peptide A-reverse (5’-AGTGCCTTCTGTTTTTAATGTGTTC-3’), 18S rRNA-forward (5′-ATGGTTGCAAAGCTGAAACT-3′) and 18S rRNA-reverse (5′-TCCCGTGTTGAGTCAAATTA-3′).

To examine the impact of phurealipids on *M. sexta* immune responses, they were dissolved in dimethylsulfoxide (DMSO) (Fisher Scientific) and used for insect injection at a final concentration of 10 µM. Larvae of *M. sexta* (Carolina Biological Supply) were reared on a wheat-germ based artificial diet (MP-Biomedicals, Solon, OH, USA) at 26 ºC with daily 18 h/ 6h light/dark photoperiods. Five 4th instar larvae were injected with *Salmonella enterica* serovar Typhimurium LT2 or PBS and 1.5 hours later were injected with 10 µM phurealipids 1, 7, 13, or the control solvent DMSO. Whole insects were snap-frozen and homogenized in TRIzol reagent (Invitrogen) 3.5 hours after treatment with phurealipids. For qRT-PRC, 5 µg of total RNA were treated with RQ1 RNase-free DNase I (Promega). Reverse transcription was performed using the Mg primer: 5-CGGGCAGTGAGCGCAACGTTTTTTTTTTT-3′ (Integrated DNA Technologies) and AMV Reverse Transcriptase (Promega). cDNA was diluted 10-fold and used as template for quantitative real time PCR (qRT-PCR). qRT-PCR was performed with SYBR® Green Supermix (Bio-Rad) on a Bio-Rad iCycler using diluted cDNAs as template. Transcript levels of cecropin, lysozyme and moricin were measured and normalized against rpS3 using the following primers: cecropin-forward (5’-GGTCAAAGGATTCGTGACGC-3’) and cecropin-reverse (5’-

TTTGATTGTCCTTTGAAAATGGCG-3’), lysozyme-forward (5’-TCGACTTGCGCCAAGAAGAT-3’) and lysozyme reverse (5’-ACGATGGGTTTCAGGACTGT-3’), moricin-forward (5’-TGAATTTCGCATTATAGGCTGTGT-3’) and moricin-reverse (5’-GAGGTATCATTTACCCGCCAC-3’), rpS3-forward (5’-ACTTCTCAGGCAAGGAGTGC-3’) and rpS3-reverse (5’-GTCACCAGGATGTGGTCTGG-3’).

Data were analyzed as described previously [22]. Briefly, Ct values were normalized by calculating the ratio between the reference gene and the gene of interest and presented as a ratio between infected versus PBS injected larvae. Data were statistically analyzed using Mixed Effect ANOVA with a Dunnett’s post-hoc test on normalized Ct values (SAS Software).

***Tribolium castaneum* embryo collection, treatment and fluorescence live imaging.** For this study, the *Tribolium castaneum* (Herbst) Vermillion White EFA-nGFP transgenic line (Sarrazin *et al.*, 2012) was crossed homozygote on one insert and reared on full grain wheat flour (#113061036, Demeter, Darmstadt, Germany) supplemented with 5% (w/v) inactive dry yeast (#62-106, Flystuff, San Diego, CA, USA) at 25°C and 70% relative humidity in a 12:00 h light / 12:00 h darkness cycle (#DR-36VL, Percival Scientific, Perry, IA, USA). For embryo collection, 300-500 adults were transferred to 10 g 405 fine wheat flour (#113061006, Demeter, Darmstadt, Germany) supplemented with 5% (w/w) inactive dry yeast and incubated for 02:00 hours at 25°C. After the incubation period, the embryos were incubated 16:00 hours at 25°C. Simultaneous dechorionation of 80-100 embryos was performed as described previously (Benton *et al.*, 2013). A drop of 300 µl 1% (w/v) low-melt agarose in PBS pH 7.4 was added to each of the inner 60 wells of a 96-well plate before attaching the embryos on top of these drops with a small paint brush one-by-one. Methoprene and HB71

were dissolved in 0.1% DMSO (v/v, #A994.1, Carl Roth, Karlsruhe, Germany) in PBS pH 7.4 (#10010-023, Gibco Life Technologies GmbH, Darmstadt, Germany) to final concentrations of 10 µM and 100 µM and 100 µl of those solutions were added to the respective wells. As controls, PBS pH 7.4 and 0.1% DMSO (v/v) in PBS pH 7.4 were used. Fluorescence live imaging started directly after the addition of the compounds and was performed on a ZEISS Cell Observer SD (Carl Zeiss, Göttingen, Germany). Embryos were illuminated with a 488 nm Argon laser (100 mW initial power, 30%), images were recorded through an EC Plan-Neofluar 10×/0.30 M27 objective (#420341-9910-000, Carl Zeiss, Göttingen, Germany) with a Rolera EM-C² camera (#01-ROL-EMC2-R-F-M-14-C, QImaging, Surrey, BC, Canada). Each embryo was imaged in 6 planes with 30 µm spacing and a 200 ms exposure time. Imaging was performed over a period of 50:00 hours with an interval of 00:15 hours. The stage speed was reduced to 10% of the maximum to prevent embryos from detaching. From the recorded planes per embryo, maximum projections were computed and embryos were rotated with their anterior pole to the top.

Statistics. Four datasets with 6 PBS control embryos, 6 DMSO control embryos and 12 embryos for each compound/concentration were recorded. Embryos that completed dorsal closure properly were scored as completing the development. Significance was determined by the two-sided student's t-test.

Chemical synthesis of glycine amides and phurealipids. If not noted differently, the chemicals were purchased in the highest purity available.

General synthetic procedure I. 1 eq of the corresponding isocyanides were dissolved in acetone (4 mL/mmol) and 2 eq of the amine were added. The reaction mixture was stirred overnight and the forming precipitate recrystallized.

General synthetic procedure II. 1 eq of the free acid was dissolved in benzene (45 mL/mmol) and 6 eq of triethylamine and 3 eq of diphenylphosphoryl azide were added. After refluxing for approximately 1h the reaction mixture was cooled to room temperature, acetone (30 mL/mmol) and ca. 10 eq of methylamine (40% in water) or ammonium hydroxide (28 – 30% in water) were added and the mixture stirred at room temperature overnight. The solvents were removed under reduced pressure and the precipitate purified with normal phase liquid chromatography or recrystallization.

General synthetic procedure III (synthesis of glycine amides). 1 eq of Boc-protected glycine was dissolved in dichloromethane (3 mL/mmol), cooled to 0°C and 1.1 eq of TBTU and 4 eq DIEA were added slowly. After stirring of 10 min at 0°C the reaction mixture was warmed to room temperature and 1.1 eq of the corresponding amine were added and stirred overnight. Upon completion of the reaction the organic phase was washed with a saturated ammonium chloride solution. The resulting aqueous phase was extracted three times with dichloromethane, then the organic phases were combined and dried over sodium sulfate. The solvents were removed under reduced pressure and the precipitate purified with normal phase liquid chromatography. The protecting group was removed quantitatively upon incubation with 4 M hydrochloric acid in dioxane (10 mL/mmol) at 0°C for several hours. Then the solvents were removed under reduced and the target compound was obtained as a hydrochloric salt.

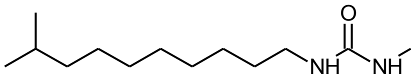

1

***N*-Methyl-*N’*-(9-methyldecyl) urea** (**1**) was obtained according to the general procedure II. The *iso*-branched fatty acid was purchased with Larodan AB (Limhamn, Sweden). Recrystallization from acetone yielded the white crystalline urea (15 mg, 0.066 mmol, 65% yield). HRMS (m/z): $[M+H]^+$ calcd. for C13H29N2O, 229.2274; found 229.2293; T_m=85°C; **^{1}H-NMR** (400 MHz, $CDCl_3$): δ 3.16 (t, J = 7.1 Hz, 2H) 2.79 (s, 3H), 1.55 – 1.47 (m, 3H), 1.30 - 1.25 (m, 10H), 1.16 (m, 2H), 0.87 (d, J = 6.6 Hz, 6H), **^{13}C-NMR** (125 MHz, $CDCl_3$): δ 158.9, 40.8, 39.1, 30.2, 29.8, 29.6, 29.3, 27.9, 27.4, 27.3, 26.9, 22.6

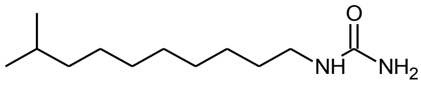

2

***N*-(9-Methyldecyl) urea** (**2**) was obtained according to the general procedure II. Recrystallization from acetone yielded the white crystalline urea (111 mg, 0.51 mmol, 86% yield). HRMS (m/z): $[M+H]^+$ calcd. for C12H27NO, 215.2117; found 215.2132; T_m=94°C **^{1}H-NMR** (400 MHz, $CDCl_3$): δ 3.11 (t, J = 7.1 Hz, 2H), 1.57 – 1.46 (m, 3H), 1.28 - 1.26 (m, 10H), 1.20-1.15 (m, 2H), 0.90 (d, J = 6.6 Hz, 6H) **^{13}C-NMR** (75 MHz, $CDCl_3$): δ 160.1, 38.9, 30.8, 30.1, 29.8, 29.5, 29.3, 27.8, 27.5, 27.3, 26.8, 22.5

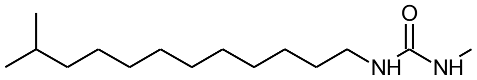

3

***N*-Methyl-*N’*-(11-methyldodecyl) urea** (**3**) was obtained according to the general procedure II. The *iso*-branched fatty acid was purchased with Sigma Aldrich (Germany). Recrystallization from acetone yielded the white crystalline urea (4.2 mg, 0.016 mmol, 25% yield). HRMS (m/z): $[M+H]^+$ calcd. for C15H33N2O, 257.2587; found 257.2594; **^{1}H-NMR**

(400 MHz, $CDCl_3$): δ 3.15 (t, J = 7.1Hz, 2H), 2.79 (s, 3H), 1.57 – 1.45 (m, 3H), 1.33 - 1.26 (m, 14H), 1.18 - 1.13 (m, 2H), 0.88 (d, J = 6.7 Hz, 6H)

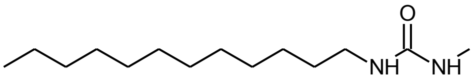

4

***N*-Methyl-*N'*-dodecyl urea** (**4**) was obtained according to the general procedure II. Recrystallization from acetone yielded the white crystalline urea (31 mg, 0.13 mmol, 52% yield). HRMS (m/z): $[M+H]^+$ calcd. for C14H31N2O, 243.2430; found 243.2465; T_m=81.1°C; **^{1}H-NMR** (400 MHz, $CDCl_3$): δ 3.14 (t, J = 7.2 Hz, 2H), 2.78 (s, 3H), 1.51-1.44 (m, 2H), 1.32-1.25 (m, 18H), 0.89 (t, J = 6.6 Hz, 3H); **^{13}C-NMR** (125 MHz, $CDCl_3$): δ 159.4, 40.4, 39.8, 31.5, 30.0, 29.2, 29.2, 29.0, 28.9, 26.6, 26.3, 22.2, 13.7

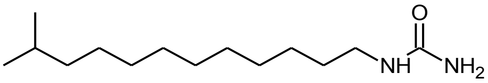

5

***N*-(11-Methyldodecyl) urea** (**5**) was obtained according to the general procedure II. The *iso*-branched fatty acid was purchased with Sigma Aldrich. Recrystallization from acetone yielded the white crystalline urea (6.6 mg, 0.027 mmol, 41% yield). HRMS (m/z): $[M+H]^+$ calcd. for C14H31N2O, 243.2430; found 243.2449; **^{1}H-NMR** (400 MHz, $CDCl_3$): δ 3.15 (t, J = 7.1Hz, 2H), 1.56 – 1.45 (m, 3H), 1.33 - 1.26 (m, 14H), 1.16 (m, 2H), 0.88 (d, J = 6.6 Hz, 6H) **^{13}C-NMR** (125 MHz, $CDCl_3$): δ 158.5, 45.6, 41.0, 39.1, 30.0, 29.9, 29.7, 29.6, 29.6, 29.3, 28.0, 27.4, 26.8, 22.7

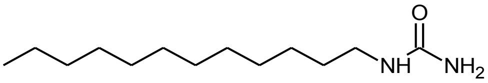

6

***N*-Dodecyl urea** (**6**)[23] was obtained according to the general procedure II. Recrystallization from acetone yielded the white crystalline urea (35.1 mg, 0,164 mmol, 65% yield). HRMS

(m/z): $[M+H]^+$ calcd. for C13H29N2O, 229.2274; found 229.2306; T_m=105.9°C; **^{1}H-NMR** (400 MHz, $CDCl_3$): δ 3.16 (t, J = 7.2 Hz, 2H), 1.54 – 1.49 (m, 2H), 1.32 - 1.27 (m, 18H), 0.89 (t, J = 6.6 Hz, 3H)

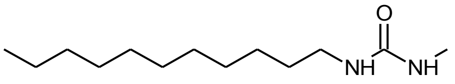

7

***N*-methyl-*N'*-undecyl urea** (**7**) was obtained according to the general procedure I. Recrystallization from acetone yielded the white crystalline urea (69 mg, 0.3 mmol, 61% yield). HRMS (m/z): $[M+H]^+$ calcd. for C13H29N2O, 229.2274; found 229.2283; T_m=88.1°C; **^{1}H-NMR** (400 MHz, $CDCl_3$): δ 3.14 (t, J = 7.2 Hz, 2H), 2.76 (s, 3H), 1.50 - 1.44 (m, 2H), 1.32-1.25 (m, 16H), 0.88 (t, J = 6.6 Hz, 3H) **^{13}C-NMR** (125 MHz, $CDCl_3$): δ 159.8, 40.4, 31.9, 30.3, 29.6, 29.6, 29.4, 29.3, 26.9, 26.8, 22.6, 14.0

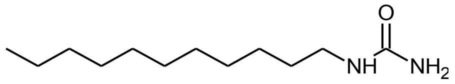

8

***N*-Undecyl urea** (**8**) was obtained according to the general procedure I. Recrystallization from acetone yielded the white crystalline urea (74 mg, 0.35 mmol, 69% yield). HRMS (m/z): $[M+H]^+$ calcd. for C12H27NO, 215.2117; found 215.2137; T_m=112.3 °C; **^{1}H-NMR** (400 MHz, $CDCl_3$): δ 3.16 (t, J = 7.2 Hz, 2H), 1.55 - 1.48 (m, 2H), 1.34-1.27 (m, 16H), 0.89 (t, J = 6.6 Hz, 3H) **^{13}C-NMR** (125 MHz, $CDCl_3$): δ 160.6, 41.0, 31.9, 29.6, 29.5, 29.4, 29.3, 29.2, 26.7, 22.7, 14.1

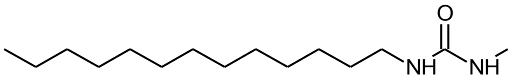

9

***N*-Methyl-*N'*-tridecyl urea** (**9**) was obtained according to the general procedure II. Recrystallization from acetone yielded the white crystalline urea (12.8 mg, 0.05 mmol, 10% yield). HRMS (m/z): $[M+H]^+$ calcd. for C15H33N2O, 257.2587; found 257.2621; **^{1}H-NMR** (400 MHz, $CDCl_3$): δ 3.14 (t, J = 7.2 Hz, 2H), 2.76 (s, 3H), 1.51 - 1.44 (m, 2H), 1.32-1.25 (m, 20H), 0.88 (t, J = 6.6 Hz, 3H)

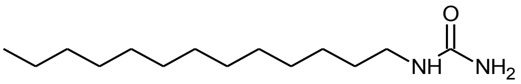

10

***N*-Tridecyl urea** (**10**)[24] was obtained according to the general procedure II. Recrystallization from acetone yielded the white crystalline urea (40.4 mg, 0.166 mmol, 34% yield). HRMS (m/z): $[M+H]^+$ calcd. for C14H30NO, 243.2358; found 243.2456; T_m=110.4°C **^{1}H-NMR** (400 MHz, $CDCl_3$): δ 3.15 (t, J = 7.2 Hz, 2H), 2.76 (s, 3H), 1.54 - 1.45 (m, 2H), 1.34-1.26 (m, 16H), 0.89 (t, J = 6.6 Hz, 3H) **^{13}C-NMR** (75 MHz, $CDCl_3$): δ 158.51, 41.0, 31.9, 30.0, 29.7, 29.6, 29.6, 29.6, 29.3, 29.3, 26.8, 22.7, 14.1

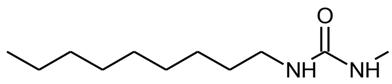

11

***N*-Methyl-*N'*-nonyl urea** (**11**) was obtained according to the general procedure II. Recrystallization from acetone yielded the white crystalline urea (53.1 mg, 0.065 mmol, 53% yield). **^{1}H-NMR** (400 MHz, $CDCl_3$): δ 3.15 (t, J = 7.1 Hz, 2H), 2.76 (s, 3H), 1.50 - 1.44 (m, 2H), 1.29-1.26 (m, 14H), 0.89 (t, J = 6.6 Hz, 3H)

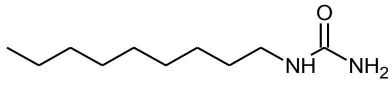

12

***N*-nonyl urea (12)**[25,26] was obtained according to the general procedure II. Recrystallization from acetone yielded yielded the white crystalline urea (18.5 mg, 0.099 mmol, 19% yield). T_m=101.1 °C; **^{1}H-NMR** (400 MHz, $CDCl_3$): δ 3.15 (t, J = 7.1 Hz, 2H), 1.50 - 1.44 (m, 2H), 1.29-1.26 (m, 12H), 0.89 (t, J = 6.6 Hz, 3H)

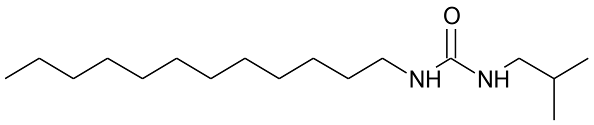

13

***N*-Dodecyl-*N*'-isobutyl urea (13)** was obtained according to the general procedure I. After recrystallization with *n*-hexane/ethylacetate 112 mg (0.39 mmol, 84%) of the white crystalline urea was obtained. HRMS (m/z): $[M+H]^+$ calcd. for C17H36N2O, 285.2906; found 285.2898; **^{1}H-NMR** (500 MHz, $CDCl_3$): δ 4.36 (sh, 2H), 3.16 (dt, J = 7.1 Hz, J = 6.5 Hz, 2H), 2.78 (d, J = 4.7 Hz, 3H), 1.57 – 1.45 (sh, 3H), 1.35 - 1.25 (sh, 10H), 0.87 (t, J = 6.6 Hz, 6H).

References

[1] H. B. Bode, D. Reimer, S. W. Fuchs, F. Kirchner, C. Dauth, C. Kegler, W. Lorenzen, A. O. Brachmann, P. Grün, *Chem. Eur. J.* **2012**, *18*, 2342–2348.

[2] A. O. Brachmann, S. A. Joyce, H. Jenke-Kodama, G. Schwär, D. J. Clarke, H. B. Bode, *Chembiochem* **2007**, *8*, 1721–1728.

[3] A. O. Brachmann, S. Brameyer, D. Kresovic, I. Hitkova, Y. Kopp, C. Manske, K. Schubert, H. B. Bode, R. Heermann, *Nat. Chem. Biol.* **2013**, *9*, 573–578.

[4] S. W. Fuchs, K. A. J. Bozhüyük, D. Kresovic, F. Grundmann, V. Dill, A. O. Brachmann, N. R. Waterfield, H. B. Bode, *Angew. Chem. Int. Ed. Engl.* **2013**, *52*, 4108–4112.

[5] S. W. Fuchs, C. C. Sachs, C. Kegler, F. I. Nollmann, M. Karas, H. B. Bode, *Anal. Chem.* **2012**, *84*, 6948–6955.

[6] D. Reimer, K. M. Pos, M. Thines, P. Grün, H. B. Bode, *Nat. Chem. Biol.* **2011**, *7*, 888–890.

[7] S. Debernard, C. Morisseau, T. F. Severson, L. Feng, H. Wojtasek, G. D. Prestwich, B. D. Hammock, *Insect Biochem.Mol.Biol.* **1998**, *28*, 409–419.

[8] S. G. Kamita, M. D. Wogulis, C. S. Law, C. Morisseau, H. Tanaka, H. Huang, D. K. Wilson, B. D. Hammock, *Biochemistry-US* **2010**, *49*, 3733–3742.

[9] B. D. Hammock, T. C. Sparks, *Anal.Biochem.* **1977**, *82*, 573–579.

[10] S. W. Fuchs, F. Grundmann, M. Kurz, M. Kaiser, H. B. Bode, *Chembiochem* **2014**, *15*, 512–516.

[11] Y.-Y. Chu, M. Nega, M. Wölfle, L. Plener, S. Grond, K. Jung, F. Götz, *PLoS Pathog* **2013**, *9*, e1003654.

[12] A. Thanwisai, S. Tandhavanant, N. Saiprom, N. R. Waterfield, P. Ke Long, H. B. Bode, S. J. Peacock, N. Chantratita, *PLoS ONE* **2012**, *7*, e43835.

[13] E. Duchaud, C. Rusniok, L. Frangeul, C. Buchrieser, A. Givaudan, S. Taourit, S. Bocs, C. Boursaux-Eude, M. Chandler, J. F. Charles, et al., *Nat. Biotechnol.* **2003**, *21*, 1307–1313.

[14] J. M. Chaston, G. Suen, S. L. Tucker, A. W. Andersen, A. Bhasin, E. Bode, H. B. Bode, A. O. Brachmann, C. E. Cowles, K. N. Cowles, et al., *PLoS ONE* **2011**, *6*, e27909–.

[15] A. Lanois, J. C. Ogier, J. Gouzy, C. Laroui, Z. Rouy, A. Givaudan, S. Gaudriault, *Genome Announcements* **2013**, *1*, e00342–13–e00342–13.

[16] P. Wilkinson, N. R. Waterfield, L. Crossman, C. Corton, M. Sanchez-Contreras, I. Vlisidou, A. Barron, A. Bignell, L. Clark, D. Ormond, et al., *BMC Genomics* **2009**, *10*, 302–302.

[17] N. Philippe, J. P. Alcaraz, E. Coursange, J. Geiselmann, D. Schneider, *Plasmid* **2004**, *51*, 246–255.

[18] S. E. Reynolds, S. F. Nottingham, *J.Insect Physiol.* **1985**, *31*, 129–134.

[19] K. Mukherjee, B. Altincicek, T. Hain, E. Domann, A. Vilcinskas, T. Chakraborty, *Appl. Environ. Microbiol.* **2009**, *76*, 310–317.

[20] K. Mukherjee, M. A. Mraheil, S. Silva, D. Muller, F. Cemic, J. Hemberger, T. Hain, A. Vilcinskas, T. Chakraborty, *Appl. Environ. Microbiol.* **2011**, *77*, 4237–4240.

[21] K. Mukherjee, T. Hain, R. Fischer, T. Chakraborty, A. Vilcinskas, *Virulence* **2013**, *4*, 324–332.

[22] J. C. Castillo, U. Shokal, I. Eleftherianos, *J.Insect Physiol.* **2013**, *59*, 179–185.

[23] T. F. Severson, M. H. Goodrow, C. Morisseau, D. L. Dowdy, B. D. Hammock, *Insect Biochem.Mol.Biol.* **2002**, *32*, 1741–1756.

[24] Y. Mido, F. Fujita, H. Matsuura, K. Machida, *Spectrochimica Acta Part A: Molecular Spectroscopy* **1981**, *37A*, 103–112.

[25] Y. Mido, S. Kimura, Y. Sugano, K. Machida, *Spectrochimica Acta Part A: Molecular Spectroscopy* **1988**, *44*, 661–668.

[26] C. C. Porter, D. C. Titus, M. J. DeFelice, *Life Sci.* **1976**, *18*, 953–959.

Table S1. Structure elucidation of the phurealipids A, C, D (**1**, **3**, **4**) and the desmethyl phurealipids B, E, F (**2**, **5**, **6**). The desmethyl derivatives **5** and **6** were only produced in the *pliB* mutant. Feeding of deuterated leucine resulted in the expected $^{2}H_{7}$-shift in **1-3** and **5** from the incorporation of L-[2,3,3,4,5,5,5,5´,5´,5´-$^{2}H_{10}$]leucine derived isovaleryl unit. Feeding of L-[*methyl*-$^{2}H_{3}$]methionine resulted in labeling of the methylated derivatives **1-3**. No incorporation of L-[2,3,4,4,4,5,5,5-$^{2}H_{8}$]valine or [2,2,3,3,3-$^{2}H_{5}$]propionate have been observed indicating only acetyl or isovaleryl as starting units.

	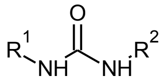		Rt [min]	$[M+H]^+$			feeding experiments	
	R^1	R^2		exp.	theo.	comp.	L-[2,3,3,4,5,5,5,6,6,6-$^{2}H_{10}$]leucine	L-[*methyl*-$^{2}H_{3}$] methionine
**1**	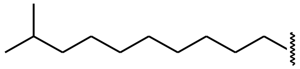	CH_3	9.6	229.2293	229.2274	$C_{13}H_{29}N_2O$	+	+
**2**	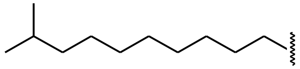	H	9.1	215.2132	215.2117	$C_{12}H_{27}N_2O$	+	-
**3**	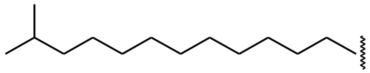	CH_3	10.8	257.2594	257.2587	$C_{15}H_{33}N_2O$	+	+
**4**	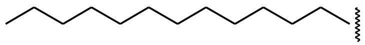	CH_3	10.3	243.2465	243.2431	$C_{14}H_{31}N_2O$	-	+
**5**	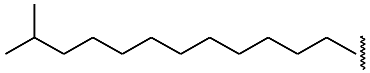	H	10.4	243.2449	243.2431	$C_{14}H_{31}N_2O$	+	-
**6**	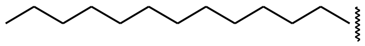	H	9.9	229.2306	229.2274	$C_{13}H_{29}N_2O$	-	-

Table S2. Overview of the biological activities of synthetic and natural phurealipids as well as their occurrence. Activity against juvenile hormone epoxide hydrolase (JHEH) and juvenile hormone esterase (JHE) from *Manduca sexta* as well as activity against *Trypanosoma brucei rhodesiense* (positive control melarsoprol IC_{50}=0.004 µg/mL), *Trypanosoma cruzi* (positive control benznidazole IC_{50}=0.498 µg/mL), *Leishmania donovani* (positive control miltefosine IC_{50}=0.174 µg/mL), and *Plasmodium falciparum* NF54 (positive control chloroquine IC_{50}=0.003 µg/mL) were performed as described previously.

	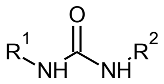		IC_{50} [µM] (% of enzyme inhibition at 100µM)		IC_{50} [µg/mL]					occurrence
	R^1	R^2	JHEH	JHE	*T. b. rhod.*	*T. cruzi*	*L. don.*	*P. falc. NF54*	L6 cells	
**1**	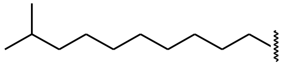	CH_3	6.5 ± 0.9 (81)	> 100 (1)	44.0	16.5	17.6	2.02	34.4	TT01 wt
**2**	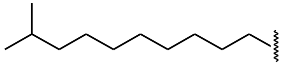	H	> 100 (0)	> 100 (2)	61.8	27.3	6.6	44.3	79.1	TT01 wt
**3**	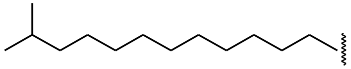	CH_3	30 ± 4 (53)	> 100 (9)	17.8	19.2	10.3	3.3	79.6	TT01 wt
**4**	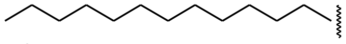	CH_3	10.7 ± 1.2 (67)	> 100 (1)	>100	47.3	94.8	15.1	>100	TT01 wt
**5**	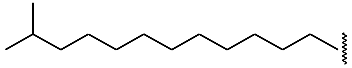	H	> 100 (9)	25 ± 4 (89)	22.8	17.5	0.9	8.1	61.1	KO *pliB*
**6**	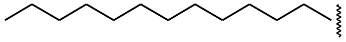	H	> 100 (8)	> 100 (5)	49.1	17.0	2.2	10.2	>100	KO *pliB* and [23]
**7**	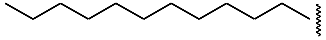	CH_3	4.3 ± 0.5 (*nd*)	> 100 (4)	62.5	19.1	18.1	8.0	40.6	synthetic
**8**	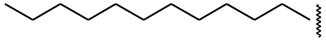	H	*nd*	*nd*	25.9	9.4	1.7	12.6	>100	synthetic
**9**	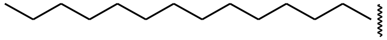	CH_3	10 ± 2 (64)	> 100 (4)	40.5	27.1	8.3	3.0	>100	synthetic
**10**	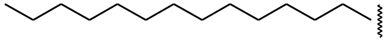	H	> 100 (2)	> 100 (9)	50.1	43.6	3.5	27.5	>100	synthetic[24]
**11**	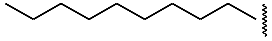	CH_3	> 100 (0)	> 100 (0)	54.6	63.8	>100	>50	>100	synthetic
**12**	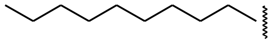	H	> 100 (6)	> 100 (39)	34.3	12.8	3.8	22.8	16.5	synthetic[25,26]
**13**	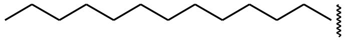	*i*Bu	2.3 ± 0.6 (*nd*)	> 100 (7)	>100	76.7	22.9	3.12	95.8	synthetic

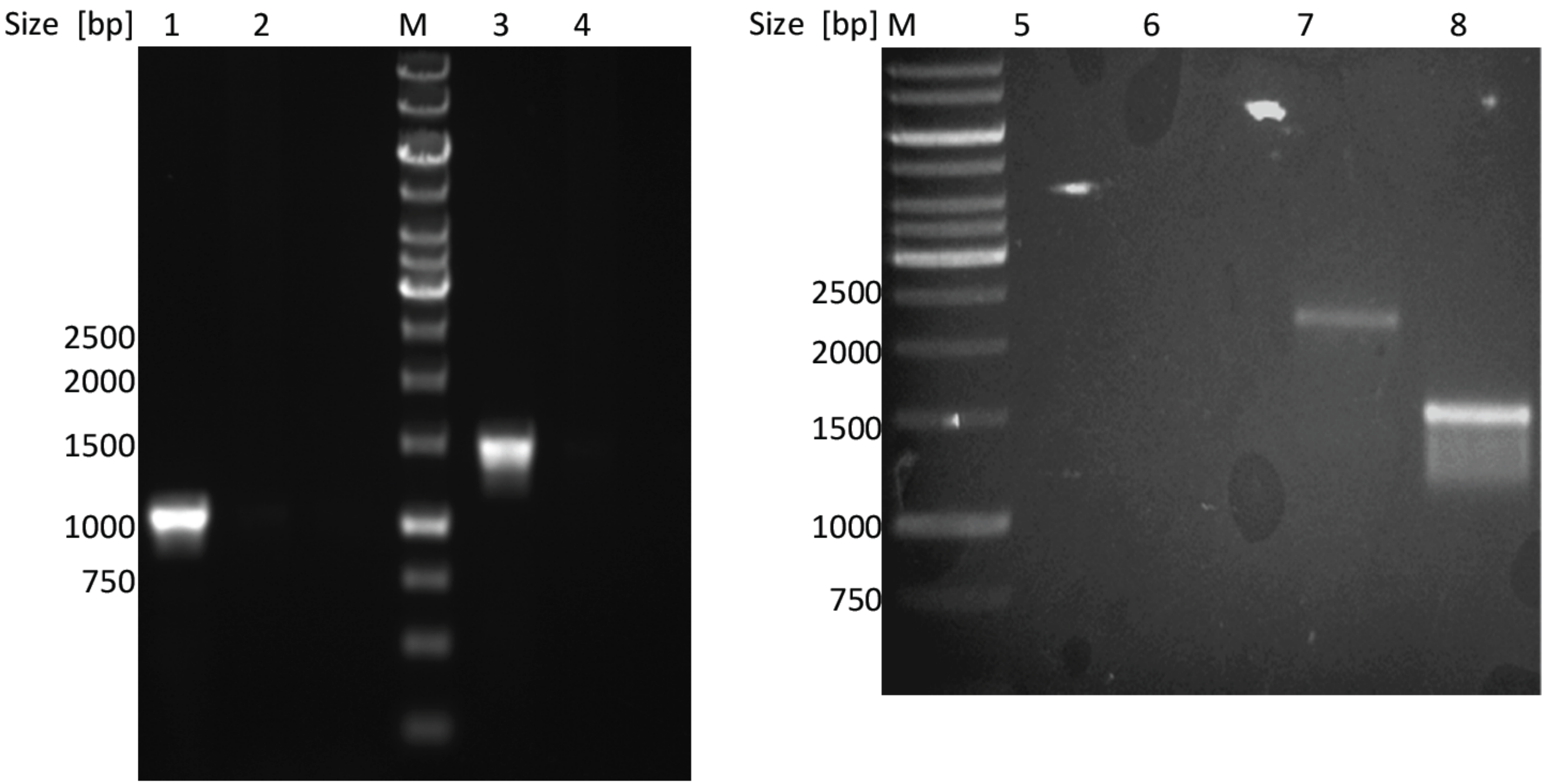

Figure S1. Genotypic Verification of the TT01-2237::cat and the TT01-2076::cat mutants by PCR
M: GeneRuler™ 1kb DNA Ladder,
1 TT01-2237::cat with pDS132fw + V_KO_plu2237_rev (1053 bp);
2 TTO1 WT with pDS132fw + V_KO_plu2237_rev;
3 TT01-2237::cat with V_KO_plu2237_fw + pDS132rv (1525bp);
4 TTO1 WT with V_KO_plu2237_fw + pDS132rv;
5 TTO1 WT with pDS132fw + V2076_Rv
6 TTO1 WT with V2076_Fw + pDS132rv
7 TT01-2076::cat with pDS132fw + V2076_Rv (~2230 bp)
8 TT01-2076::cat with V2076_FW + pDS132rv (~1550 bp)

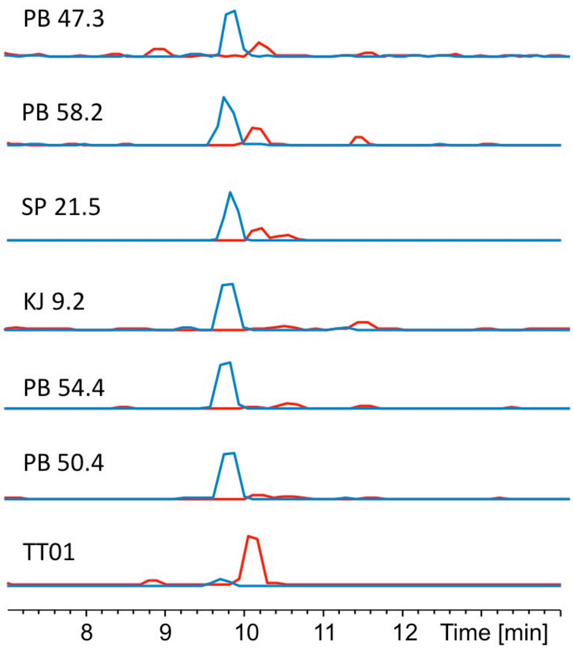

Figure S2. HPLC/MS analysis of selected strains of *P. luminescens* showing the production of different phurealipids. The EICs of **1** (red) and **2** (blue) are shown. In several cases an inversion of the amounts of produced phurealipids **1** and **2** in comparision to TT01 is observed (*Photorhabdus* PB 47.3, *Photorhabdus* PB 58.2 and *Photorhabdus* SP 21.5). Moreover, a total loss in the production of **1** can also be observed (*Photorhabdus* KJ 9.2, *Photorhabdus* PB 54.4 and *Photorhabdus* PB 50.4).

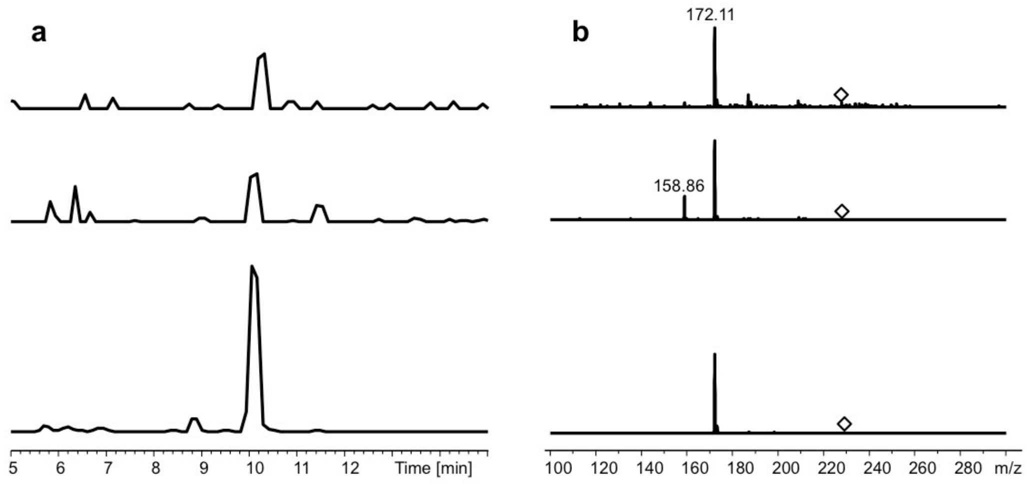

Figure S3. LC-MS/MS analysis of selected *Xenorhabdus* strains isolated from Vietnam (*Xenorhabdus* 1LC 1.2 (top) and *Xenorhabdus* DL13 red (middle)) in comparison to *Photorhabdus luminescens* TT01 (bottom). HPLC/MS chromatogram (**a**) and MS/MS analysis of the mother ion of **1** (m/z 229 $[M+H]^+$ diamond; **b**).

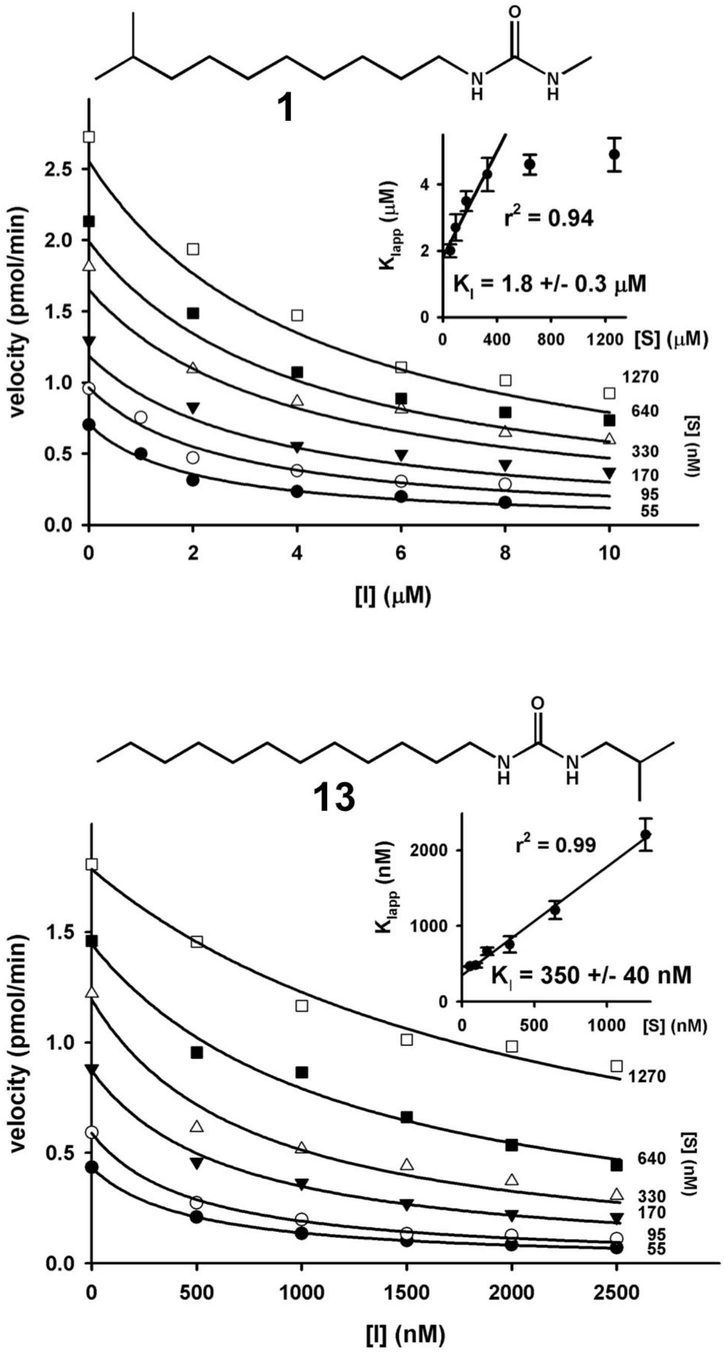

Figure S4. Determination of the K_I of compounds **1** and **13** with *Manduca sexta* JHEH using [^{3}H]-JH III as substrate. For each substrate concentration (55-1270 nM), the velocity is plotted as a function of **1** concentration (0-10 μM), allowing the determination of an apparent inhibition constant (K_{Iapp}). K_{Iapps} are plotted as a function of the substrate concentration (insert). For [S] = 0, a K_I value of 1.8 μM was found (top). For each substrate concentration of **13** (55-1270 nM), the velocity is plotted as a function of **13** concentration (0-2.5 μM), allowing the determination of an apparent inhibition constant (K_{Iapp}). K_{Iapps} are plotted as a function of the substrate concentration (insert). For [S] = 0, a K_I value of 350 nM was found (bottom).

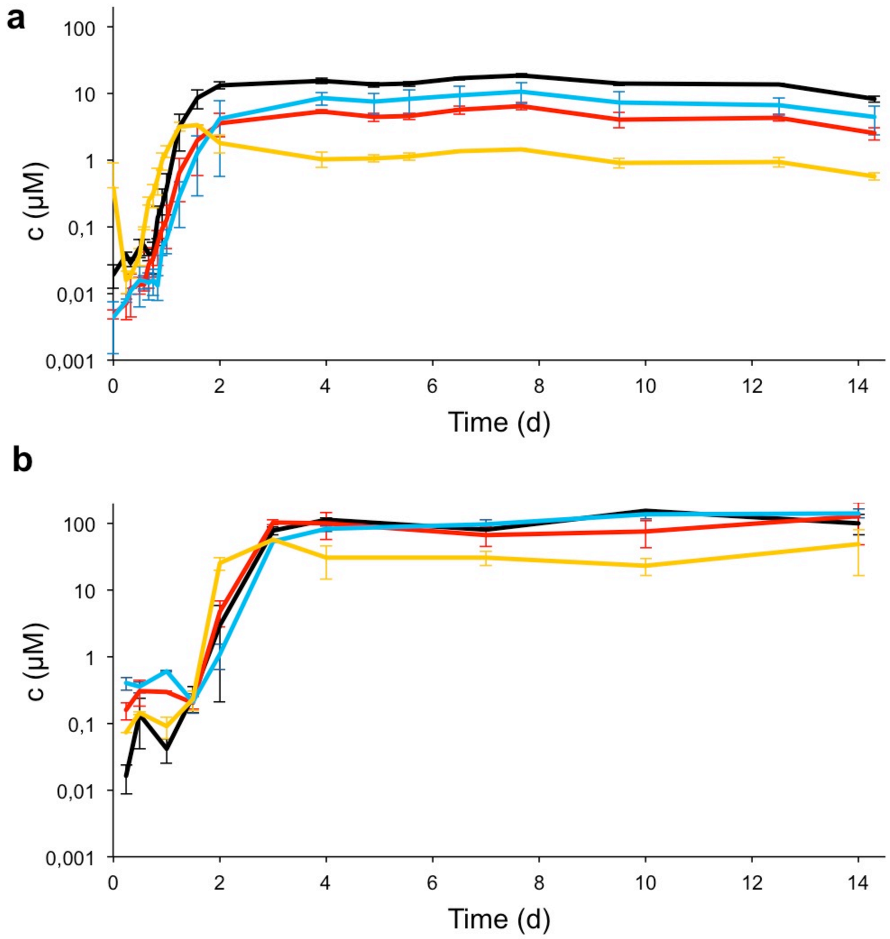

Figure S5. Production kinetics of phurealipids of TT01 cultivated in LB broth (a) and after the injection of the bacteria in larvae of *Galleria mellonella* (b) based on LC-MS measurements. An increase in production of the phurealipids **1** (black), **2** (orange), **3** (blue) and **5** (red) can be observed after the injection in the insect. In order to determine an absolute concentration of the phurealipids, a calibration curve was compiled based on five different concentrations of the synthetically obtained **1** measured as triplicates. The absolute and maximal amount of the main compound **5** in *G. mellonella* was 208 µM. The absolute and maximal amount of the main compound **1** in LB broth was 19 µM.

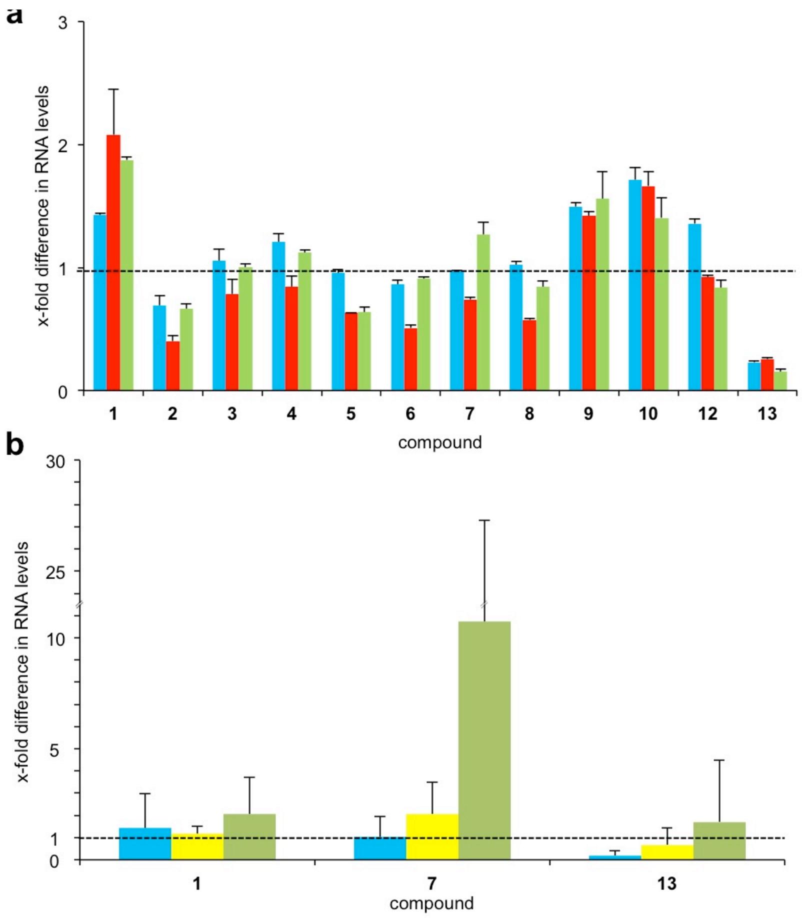

Figure S6. a, Level of transcription of the immune responsive genes responsible for the production of lysozyme (blue), gallerimycin (red) and moricin (green) in *Galleria mellonella* after injection of *S. entomophila* following injection of phurealipids. **b**, Level of transcription (relative to a DMSO control) of the immune responsive genes responsible for the production of lysozyme (blue), cecropin (orange) and moricin (green) in *Manduca sexta* after injection of *Salmonella enterica* serovar Typhimurium following injection of selected phurealipids.

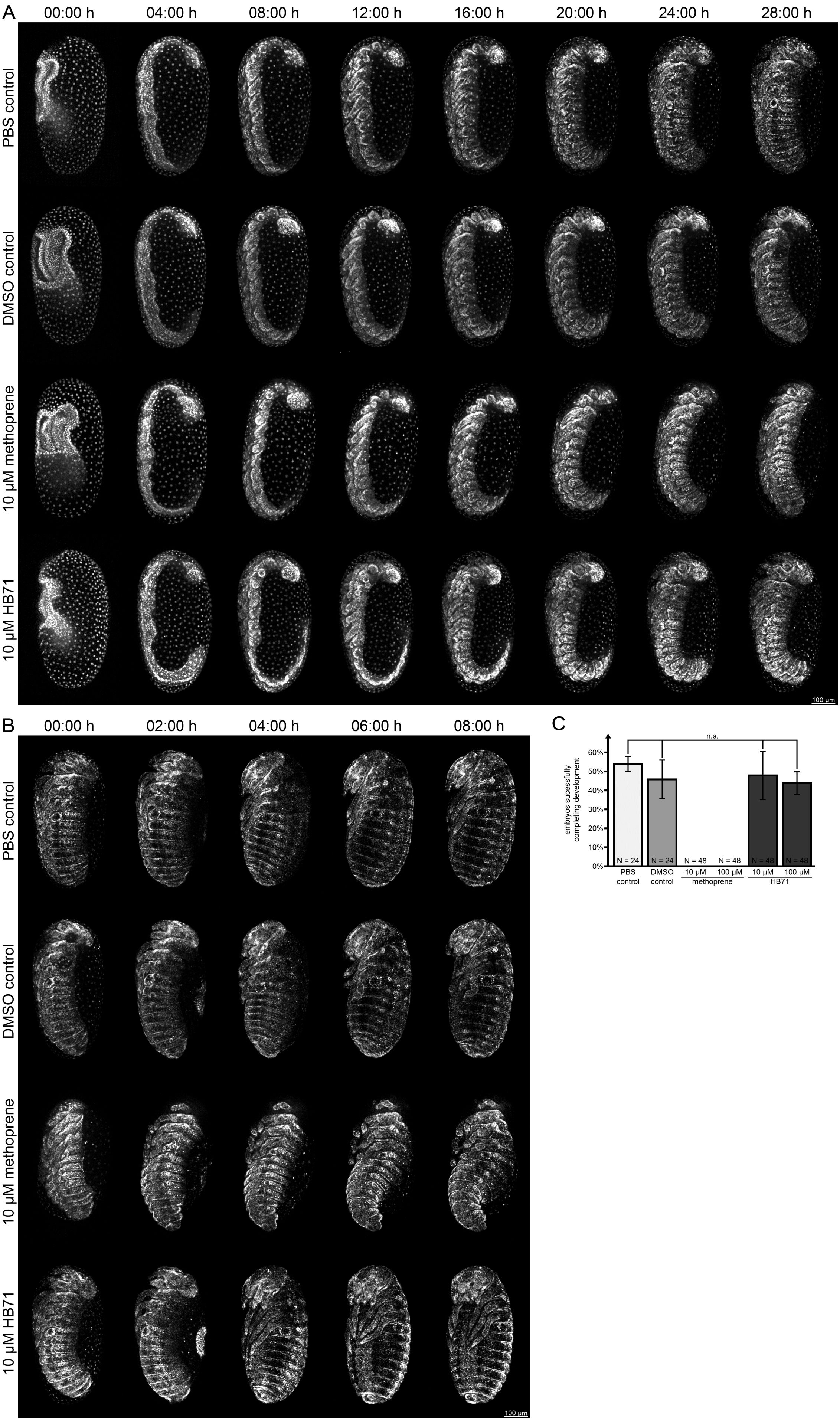
A
00:00 h
04:00 h
08:00 h
12:00 h
16:00 h
20:00 h
24:00 h
28:00 h
PBS control
DMSO control
10 µM methoprene
10 µM HB71
100 µm
B
00:00 h
02:00 h
04:00 h
06:00 h
08:00 h
PBS control
DMSO control
10 µM methoprene
10 µM HB71
100 µm
C
n.s.
embryos sucessfully completing development
60%
50%
40%
30%
20%
10%
0%
N = 24
N = 24
N = 48
N = 48
N = 48
N = 48
PBS control
DMSO control
10 µM
100 µM
methoprene
10 µM
100 µM
HB71

Figure S7. Fluorescence live imaging of *Tribolium castaneum* embryogenesis under the effects of methoprene and **13** (HB71). (**A**) Gastrulation (00:00 h), germ band elongation (04:00-08:00 h) and germ band retraction (12:00-28:00 h). In this development period, no influence of 10 µM methoprene and 10 µM **13** on morphogenesis are visible. (**B**) Dorsal closure: Embryos treated with 10 µM methoprene failed to complete dorsal closure properly. **13** did not affect the dorsal closure process, as the embryo does not differ from the control embryos. (**C**) Quantification of fluorescence live imaging: Approximately half of the embryos in the PBS and DMSO control were able to complete development. Both tested concentrations of methoprene did result completely in development aberrations, while successful development under the two tested concentrations of **13** (HB71) did not significantly differ from the controls. Error bars show S.E.M. n.s., not significant.

Data analysis (in % living *T. castaneum* embryos) of the Fluorescence live imaging experiments. MP (methoprene).

Date	Exp.	PBS	0.1% DMSO	MP (100 µM)	**13** (100 µM)	MP (10 µM)	**13** (10 µM)
08.08.	1	50	66.7	0	58.3	0	58.3
12.08.	2	50	50	0	41.7	0	41.7
27.08.	3	50	16.6	0	8.3	0	33.3
05.09	4	66.7	50	0	66.7	0	58.3
Mean		54.1	45.8	0	43.8	0	47.9
S.E.		8.3	21.0	0	25.8	0	12.5

Supplementary Movie 1. *Tribolium castaneum* embryogenesis under the effects of 10 µM methoprene and 10 µM **13** (HB71) from 00:00 h to 50:00 h with an interval of 00:15 h between the time points. The embryo treated with 10 µM methoprene failed to complete dorsal closure properly, resulting in a sigmoid shape. **13** did not affect the dorsal closure process, as the embryo does not differ from the control embryos. Frame rate is 5 frames per second.

(see separate file)
